# Penetrating gunshot wounds to the penis: a case report of combat patient injured in the war in Ukraine

**DOI:** 10.1186/s12245-023-00481-5

**Published:** 2023-02-03

**Authors:** Sergiy Golovko, Rostislav Gybalo, Igor Lurin, Igor Taraban, Artem Kobirnichenko, Vitalii Ganiuk, Maksym Gorobeiko, Andrii Dinets

**Affiliations:** 1 Urology Clinic of National Military Medical Clinical Centre, Main Military Clinical Hospital, Kyiv, Ukraine; 2 Department of Surgery, National Military Medical Clinical Centre, Main Military Clinical Hospital, Kyiv, Ukraine; 3 National Academy of Medical Sciences of Ukraine, Kyiv, Ukraine; 4 State Institution of Science, Research and Practical Center of Preventive and Clinical Medicine, State Administrative Department, Kyiv, Ukraine; 5 Department of Surgery #1, Kharkiv National Medical University, Kharkiv, Ukraine; 6 Intensive Care Unit (For Surgical Patients), National Military Medical Clinical Centre, Main Military Clinical Hospital, Kyiv, Ukraine; 7 Department of Surgery, Institute of Biology and Medicine, Taras Shevchenko National University of Kyiv, Demiїvska 13, Kyiv, 03039 Ukraine

**Keywords:** Gunshot trauma, Penis, Urethral injury, War-related penile injury, War in Ukraine

## Abstract

**Background:**

The current war in Ukraine is associated with frequent applications of multiple-launch rocket systems and cruise missiles as well as other various high-energy weapons to cause severe injuries in military personnel including abdomen wounds, vascular injury, and limb amputations as well as genitourinary trauma. The aim of this report is to demonstrate a case of successful penile salvage by restoring its function in a combat patient with gunshot genitourinary trauma in conditions of an interrupted supply of medical equipment.

**Case presentation:**

We describe a case of a 48-year-old male patient with a combined shrapnel gunshot wound to the penis with damage to the urethra and combined injury to the soft tissues of the left thigh. Several hours after the injury, the patient underwent primary surgical debridement of the left thigh, ligation of the great saphenous vein of the thigh, primary sutures on the penile urethra and navicular fossa, suturing of the rupture of the head and penis, drainage of the wound, catheterization of the bladder, and epicystostomy. An artificial erection was performed intraoperatively.

The urethral catheter was removed 3 weeks after urethral suturing (May 4, 2022). The epicystostomy was removed 5 months after the injury (August 4, 2022) and 2 days after the restoration of spontaneous urination. At the follow-up of 7 months after the injury, the patient has normal urination with minor urinary dribbling, sufficient erection, and ejaculation.

**Conclusions:**

We have shown that in a case of gunshot wounds to the penis and hanging part of the urethra, even in the presence of combined severe purulent lesions of non-urological localizations, it is possible to perform a primary reconstruction of urogenital injuries using a primary urethral suture and applying a negative pressure device. Findings from this case report shed new light on the management of penile gunshot injury in ongoing warfare as well as provide evidence of the possibility to perform adequate management for penile injury in conditions of limited medical resources, violation of international humanitarian law, and under frequent strikes of high-energy weapons.

## Background

Armed conflicts and wars are associated with various gunshot trauma in military personnel or civilians, including penile injuries [1]. The war between Russia and Ukraine has started in 2014 as a hybrid warfare, followed by an active invasion phase since February 24, 2022 [2–4]. Since then, the aggressor uses various high-energy weapons, causing severe injuries. These kinds of injuries are diagnosed with typical or atypical presentations, representing in some cases clinical challenges for the Ukrainian military and civil medical doctors as showed in our previous reports [2, 5, 6]. The current war in Ukraine is associated with frequent applications of multiple-launch rocket systems and cruise missiles as well as other various high-energy weapons to cause severe injuries in military personnel including abdomen wounds, vascular injury, and limb amputations as well as genitourinary trauma [3, 5, 7–9]. The management of the latter is well-described in military and civil cohorts; however, little is known about the clinical features of penile gunshot trauma in the war in Ukraine. It is also worth mentioning that Ukrainian medical facilities at all levels of medical care are at high risk of missile attacks by the Russian army, which is demonstrated in frequent violation of the humanitarian international law including war-related conventions [10]. Under the abovementioned conditions, genitourinary trauma, and specifically gunshot injuries to the penis, it is difficult to treat such patients as well as to achieve excellent results, which are normal urination, erection, and ejaculation.

The aim of this report is to demonstrate a case of successful penile salvage by restoring its function in a combat patient with gunshot genitourinary trauma in conditions of an interrupted supply of medical equipment in the war in Ukraine.

## Case presentation

The wounded Ukrainian man 48 years old received a fragmentation mine-explosive wound from a cluster munition after artillery shelling on the battlefield area near Kyiv (i.e., the capital of Ukraine) on March 15, 2022.

The management of the patient was performed according to the medical military doctrine of Ukraine, which included 5 levels of medical care as described previously [3]. In the first few hours after the injury, the patient was diagnosed with a combined gunshot shrapnel wound of the soft tissues of the left thigh and penis, and hemorrhagic shock stage II and received the first medical aid at level I of medical care. The first medical aid was provided by stopping the bleeding and applying aseptic pressure bandages to the wounds after the application of local anesthesia.

At level II of medical care (i.e., specialized surgical care), the patient’s condition was assessed as moderate to severe due to the nature of the injury, blood loss, pain syndrome, and psychotrauma.

Local examination showed a gunshot wound to the left inguinal region with a massive soft-tissue defect, a section of the head and body of the penis with a defect of 1/3 of the circumference with partial damage to the albuginea of the cavernous bodies, and a complete rupture of the urethra.

The wound entry site was on the anterior surface of the upper third of the left thigh, 4×6 cm in size, with jagged edges, and bled venously. The wound channel was presented from top to bottom, from front to back of the penis. The opening on the posterior surface of the lower third of the left thigh was 6×8 cm in size, bled by capillary action. The bottom of the wound was represented by the muscles of the medial group of the left thigh. Neither rectal nor prostate pathology was detected.

**Fig. 1:**
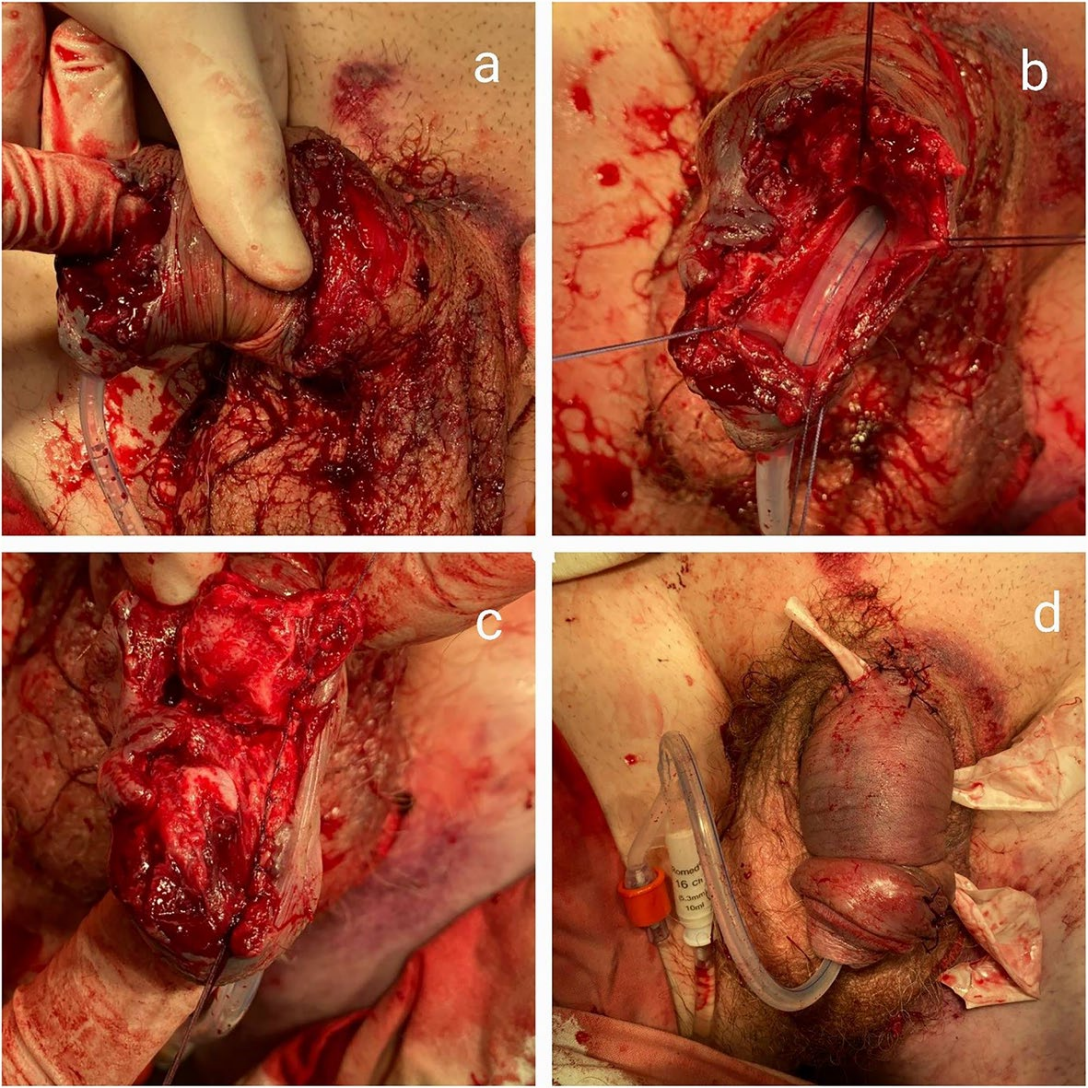
Illustration of the stages of primary surgical treatment of penile wound: revision of the wound channel (**a**), the damaged area of the penile section of the urethra is taken on the holders (**b**), the integrity of the penile urethra was restored with vicryl suture 4/0 (**c**), and the appearance of the wound after suturing and drainage (**d**)

Several hours after the injury, the patient underwent primary surgical debridement of the left thigh, ligation of the great saphenous vein of the thigh, primary sutures on the penile urethra and navicular fossa, suturing of the rupture of the head and penis, drainage of the wound, catheterization of the bladder, and epicystostomy (Fig. 1). In order to control the integrity of the corpora cavernosa, an artificial erection was performed intraoperatively.

To improve wound healing, a hermetic vacuum dressing was applied to the wound of the left thigh using a vacuum-assisted closure (VAC) device and creating a negative pressure of 100 mm Hg.

To prevent wound complications (infections, seromas, hematomas, etc.), a polyurethane sponge was fixed to the skin of the penis along the line of skin sutures with attachment to the VAC system and a negative pressure of 125 mm Hg.

**Fig. 2:**
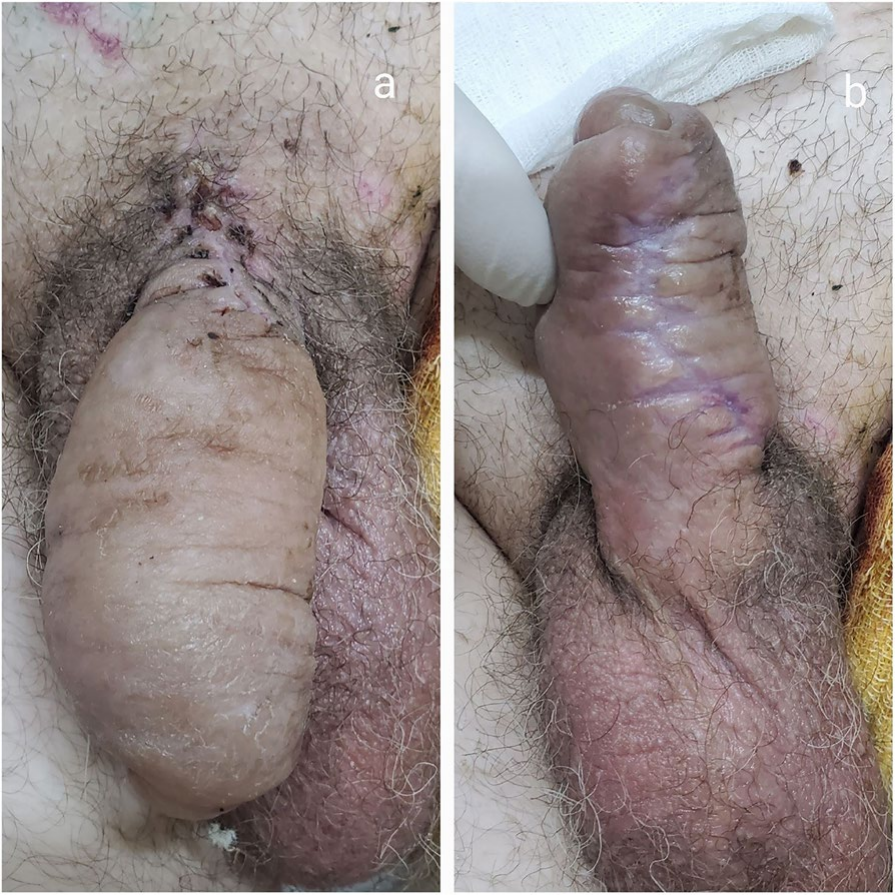
The appearance of the wound of the penis 21 days after the injury. Primary wound healing of the dorsal (**a**) and ventral (**b**) surface of the penis

At level IV of medical care (i.e., specialized surgical care), the following treatment was applied: antimicrobial therapy with ceftriaxone 2 mg, painkillers (Dexalgin, Ketonal), and an antitetanus serum (3000 IU). At the further stages of surgical treatment 2 days after the injury (March 17–19, 2022), the patient was undergone for repeated surgical debridement of the femoral wound followed by the application of the “pulsed lavage” system and installation of the VAC system (installed on March 19, removed on March 22). The healing wound of the penis occurred by primary intention (Fig. 2), and the removal of drains was performed on the 5th day after the injury. The Doppler ultrasound and uroflowmetry were performed to control the blood supply. Restoration of the spontaneous erection was observed on the 18th day after injury. The urethral catheter was removed 3 weeks after urethral suturing (May 4, 2022). The epicystostomy was removed 5 months after the injury (August 4, 2022) and 2 days after the restoration of spontaneous urination.

**Fig. 3:**
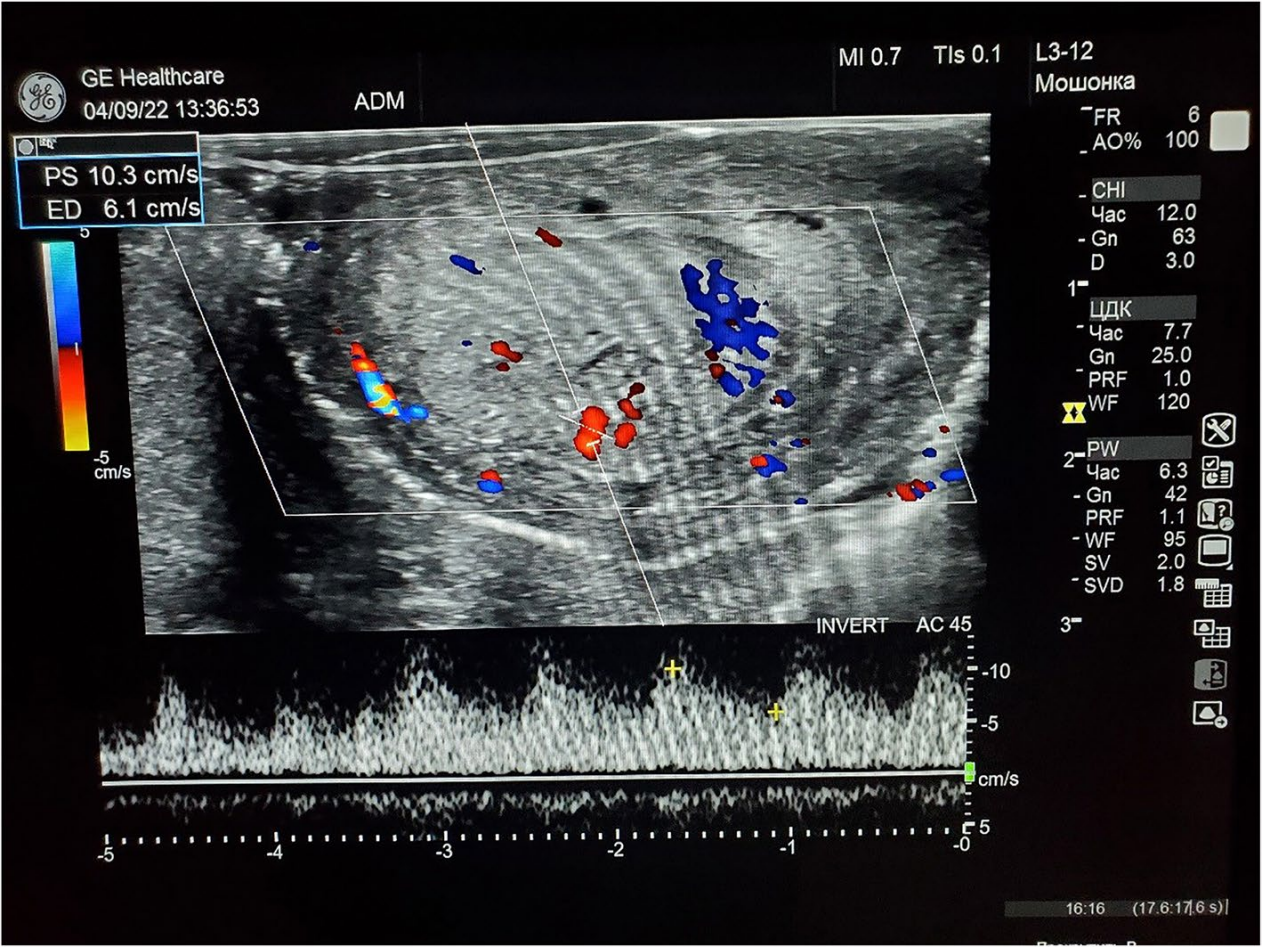
Doppler ultrasonography of the arteries of the penis: determines the main blood flow in the arteries of the corpora cavernosa (MSC 16–24 cm/s, Ri 0.73–0.77); in the head small arteries with collateral blood flow MSC 0.7 ml/s, Ri 0.62

**Fig. 4:**
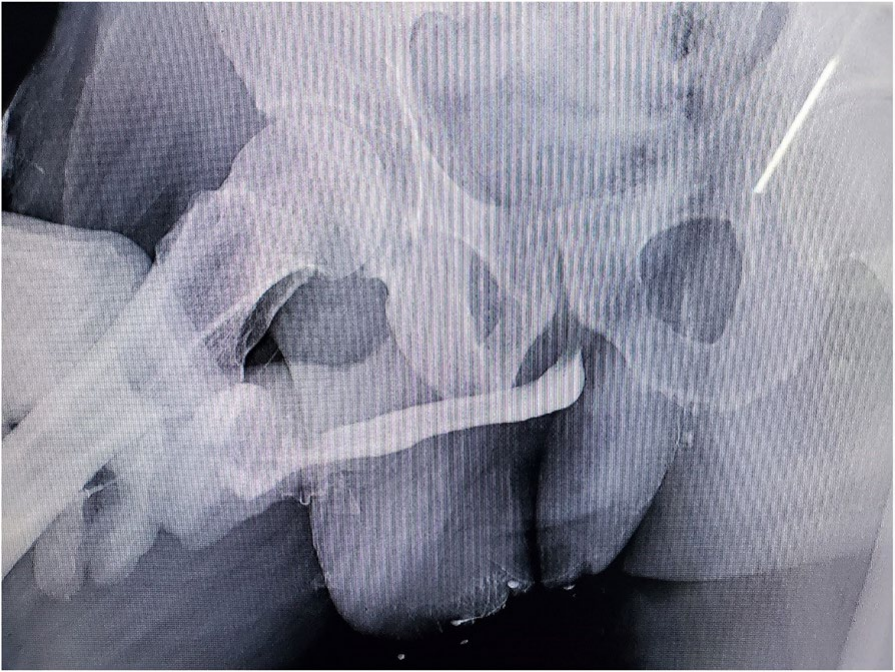
Photograph of retrograde pericatheter urethrogram 21 days after the injury. The urethra was passable, and no extravasation of the contrast agent was detected

According to the Doppler ultrasonography of the vessels of the penis, the peak systolic blood flow velocity was 16–24 ml/s (Fig. 3), indicating a normal parameter; antegrade diastolic blood flow was not determined; and resistance index was 0.83, which is normal. According to the results of pericatheter urethrography, the urethra was passable, and extravasation of the contrast medium was not detected (Fig. 4). According to the results of uroflowmetry, the maximum urination rate *Q*_max_ was 12.9 ml/s, which is normal. At the follow-up in November 2022 (7 months after the injury), the patient has normal urination with minor urinary dribbling, sufficient erection, and ejaculation.

## Discussion

In this study, we reported an example of the successful management of a combat patient with a severe gunshot injury to the penis in the war in Ukraine. This case report is provided evidence of the possibility to perform urological aid in the levels of medical care in conditions of limited medical recourses [11] and under the high risk of missile strikes on civil and combat medical facilities, other violations of international humanitarian rules by Russia, including damage of critical infrastructure and civil power stations in Ukraine [10]. However, military and civil surgeons at levels of medication can provide appropriate management of such a severe penile injury even in difficult conditions.

According to published series, damage to the organs of the urinary system during combat conflicts occurs in 0.5–4.2% of cases of damage to all the organs and systems. Among them, penile injuries account for 10–16% and, as a rule, have a combined character, which determines the severity of this type of injury [8, 12, 13]. In 82.2% of cases, such wounds are due to mine-explosive injury. The result of a bullet or a fragment hitting the body is the formation of a temporary pulsating cavity [14]. Depending on the ballistic properties, 3 zones are formed in the wound channel: the zone of the wound channel, primary necrosis, and molecular contusion, which is associated with severe damage to the tissues [15].

The incidence of gunshot wounds to the penis during the military conflict in Iraq (4%) [16] was slightly lower compared to the data obtained during the conflict between Croatia and Bosnia (9.2-9.5%) [12, 17]. The data from Issam S. Al-Azzawi et al. showed that urethral injuries accounted for 5.6% of genitourinary system injuries. These data were similar to those reported during the Vietnam war (5.6%) [16] and Croatia/Bosnia (3.5–5.3%) [12, 17].

Our data is in line with others, showing satisfactory cosmetic results along with a normal erection in 31% of patients, and a slight curvature of the penis was observed in 31% of patients. However, the authors reported severe angulation and erectile dysfunction in 19% of cases with damage to the corpora cavernosa [9].

A gunshot wound to the penis is accompanied by bleeding, which correlates with the size and depth of the wound, and is often significant in cases of damage to the cavernous bodies. Abundant blood supply to the tissues of the penis and especially the cavernous bodies is the main reason for the formation of massive bleeding with the formation of large hematomas [18]. In such situations, the blood imbibes the subcutaneous tissue, blocks the lymphatic vessels, resulting in swelling of the skin, and the skin itself acquires a blue-purple color.

Our experience shows that in receiving a penile injury in the first place, it is important to take a thorough medical history and conduct a detailed examination of the site of injury. The localization and size of the wound defect are being visually established, and the intensity of bleeding should immediately be stopped by available methods at the stage of first medical aid. The amount of damage is established, and options for medical care are evaluated. The optimal volume of assistance is the timely delivery of the injured person to the stage of specialized surgical care. In a clinic, it is necessary to conduct an optimal diagnostic algorithm using X-ray, ultrasound techniques, and pharmacological tests. The exact establishment of the final diagnosis determines the optimal tactics of the planned intervention. The main stage of treatment is the adequate implementation of the primary surgical treatment.

In case of damage to the urethra, which we have described, we recommend performing a one-stage surgical reconstruction. This is because when receiving such injuries in the conditions of the defense of urban megacities, there is the possibility of rapid evacuation to a specialized urological department of a military hospital. Performing an epicystostomy and a delayed repair of the urethra may be the best option in cases of the unstable condition of the injured person or the absence of relevant experience from a urologist [16].

Our experience shows that an important component of a successful diagnosis is adherence to the following diagnostic algorithms:

1. Radiography of the pelvic organs
2. Retrograde urethrography
3. Intraoperative artificial erection (allows for diagnosis of damage to the cavernous bodies)
4. Doppler sonography of the penis

Doppler ultrasonography of the penis is useful to identify the presence and extent of damage to the corpora cavernosa. It should be taken into account that a bullet or fragments are not sterile and may have injured a tissue (i.e., penis) [16]. Therefore, the implementation of antimicrobial prophylaxis for gunshot wounds is mandatory. In addition, we believe that constant supervision and treatment by a psychologist are also very important.

Timely diagnosis and proper treatment can help to avoid potentially dangerous long-term physical, psychological, and functional consequences of traumatic injuries to the penis. Timely restoration of the integrity of the penis ensures the rapid restoration of sexual and reproductive function.

The peculiarity of assisting this wounded man in the conditions of hostilities was that they were carried out near the metropolis and had well-established logistics; after providing primary medical care, the wounded soldier was quickly taken to the specialized urological department of the military hospital. This is what has allowed us to conduct a comprehensive examination and one-stage primary surgical treatment with the primary restoration of the urethra. In addition, the timely provision of complex rehabilitation therapy, using modern treatment methods (e.g., VAC therapy), has ensured relatively rapid wound healing and optimal functional results.

## Conclusion

We have shown that in a case of gunshot wounds to the penis and hanging part of the urethra, even in the presence of combined severe purulent lesions of non-urological localizations, it is possible to perform a primary reconstruction of urogenital injuries using a primary urethral suture and applying a negative pressure device. Findings from this case report shed new light on the management of penile gunshot injury in ongoing warfare as well as provide evidence of the possibility to perform adequate management for penile injury in conditions of limited medical resources, violation of international humanitarian law, and under frequent strikes of high-energy weapons.

## Data Availability

All data regarding this case report has been reported in the manuscript. Please contact the corresponding author in case of requiring any further information.
